# The Influence of Maceration and Flavoring on the Composition and Health-Promoting Properties of Roasted Coffee

**DOI:** 10.3390/nu16172823

**Published:** 2024-08-23

**Authors:** Joanna Grzelczyk, Grażyna Budryn, Krzysztof Kołodziejczyk, Joanna Ziętala

**Affiliations:** 1 Institute of Food Technology and Analysis, Faculty of Biotechnology and Food Sciences, Lodz University of Technology, 90-537 Lodz, Poland; grazyna.budryn@p.lodz.pl (G.B.); 237523@edu.p.lodz.pl (J.Z.); 2 Department of Sugar Industry and Food Safety Management, Faculty of Biotechnology and Food Sciences, Lodz University of Technology, 90-537 Lodz, Poland; krzysztof.kolodziejczyk@p.lodz.pl

**Keywords:** antioxidant activity, phenolic acids, organic acids, coffee flavoring

## Abstract

Over the years, many methods of refining green beans have been developed, including maceration aimed at enriching the coffee aroma and improving the overall quality. This study aimed to evaluate the influence of different methods of maceration (fruit and wine) and the addition of food flavors to coffee beans on antioxidant activity, caffeine, phenolic and organic acid content, as well as health-promoting properties. This research showed that the use of the maceration in melon and apple fruit pulp (100 g of fruit pulp per 100 g of green coffee, incubated for 24 h, coffee roasting at 230 °C, control trial roasted coffee) ensured the highest polyphenol (hydroxycinnamic acids and their esters—chlorogenic acids) content (in melon pulp—13.56 g/100 g d.b. (dry bean); in apple pulp—13.22 g/100 g d.b., *p* < 0.05 (one-way ANOVA)) and antioxidant activity. Melon (92.11%, IC50 = 3.80 mg/mL extract) and apple (84.55%, IC50 = 4.14 mg/mL) showed the highest α-amylase (enzyme concentration 10 μmol/mL) inhibition activity (0.5 mg/mL for both fruits). The addition of food flavors reduced the total content of chlorogenic acids to the range of 4.64 to 6.48 g/100 g d.b. and increased the content of acrylamide and 5-HMF, which positively correlated with a low antioxidant potential compared to the macerated samples and the control. Studies have shown that coffee macerated in the pulp of melon and apple fruit, due to its great potential to inhibit α-amylase in vivo, may have a preventive effect on type II diabetes. This study complements the current knowledge on the potential health-promoting properties of coffee flavored using different methods; further research should include more advanced models for testing these health-promoting properties. Statistical analysis was based on the determination of the average values of six measurements and their standard deviation, as well as on the one-way ANOVA (analysis of variation) and the Pearson correlation coefficient, using Statistic 10.0 software. The significance was defined at *p* ≤ 0.05.

## 1. Introduction

Due to the increasing interest of consumers in flavored coffee products, many methods and practices used by producers have been developed in this regard. Coffee flavoring initially involved the addition of artificial coffee aromas or natural coffee oil, the purpose of which was to enrich the typical coffee aroma. The next practice was using aromas with quite different food fragrance notes [1].

Traditional methods that naturally improve coffee aroma include, for example, roasting coffee at different temperatures, using various techniques for drying green coffee beans, and mixing several types of coffee [1,2,3,4]. Recent years have brought progress in food technology, and modernized production methods allow for the development of more ecological, healthy, and tasty products [2,3]. Consumers expect to buy coffee beans of the highest quality and content of health-promoting compounds [2,3]. Roasted coffee reveals one of the most complex aromas (about 1000 different compounds), like roasted cocoa or wine, although formed by different groups of volatiles. Therefore, one of the most common flavored coffees is that with chocolate aroma. On the other hand, the aroma of wine can enrich coffee beans with fruity characteristics, making them milder in taste [4]. Individual volatile compounds (i.e., pyrazines, aldehydes, ketones) are responsible for specific aroma aromas [5,6,7,8]. The sugar content in green coffee has a positive effect on the aromatic properties, causing the formation of furans and pyrroles with a caramel aroma. In turn, lactic and acetic acids add a fruity and winey aroma to coffee, as do the presence of alcohols and alpha-damascene [9,10,11]. Hydroxycinnamic acids and their esters are chemical compounds that ensure antioxidant properties in coffee. Another critical component of coffee beans is dietary fiber, which intensifies intestinal peristalsis. In green coffee beans, it is cellulose and hemicellulose, which are found in larger amounts in Arabica species compared to the content in Robusta. In roasted beans, the fiber fraction also includes Maillard reaction products. Arabica contains up to 3% more lipids than Robusta. The lipids contribute to the aroma of roasted beans by forming some ketones and aldehydes. The lipids contained in coffee consist of include sterols and tocopherols next to the most abundant triacylglycerols [12].

Coffee accompanies the consumer every day. It is consumed most often due to its energy effect. Other reasons include drinking coffee, which is popular in company at meetings or at work. A smaller proportion of consumers still consume coffee due to its health-promoting qualities. If the consumer decides to drink coffee for health reasons, they have expectations regarding its health claims. The process of flavoring coffee can enrich coffee with valuable ingredients, such as vitamins, or micro- and macro-elements, and include supplying health claims on the packaging [13,14]. On the other hand, for coffee connoisseurs, the highest quality of coffee is important while maintaining taste sensations. Therefore, consumers expect useful information such as the procedure for appropriate preparation of coffee depending on the species, the species and place of coffee bush cultivation, the date of coffee bean harvesting, and methods of pre-treatment [15]. An especially important aspect for today’s consumer is whether coffee is grown ethically [16].

One of the most frequently chosen coffees by consumers is old brew. Thanks to this method, the coffee has a unique flavor, and the coffee is characterized by a sweet, floral, and fruity taste. The noticeable bitterness and acidity are low. Consumers are increasingly trying flavored coffees. This is due to aroma marketing. Companies create coffee aromas that we associate with pleasant moments or peace. They use a subconscious technique to “program” our decisions. Only a true coffee connoisseur can appreciate the natural note of coffee [17].

Demanding consumers often look for new products and do not become permanently attached to even the best ones. In the field of coffee brands, many flavored coffees have appeared, giving the opportunity to try new flavors. The most natural improvement in aroma can be achieved by influencing the composition of the beans before roasting [18]. Mechanical drying of green beans after removing the coffee cherry pulp, compared to natural drying, reduces the level of desirable aromatic compounds after roasting. Wetting the beans before roasting or steaming during the process improves the aroma [19,20,21]. Therefore, one of the methods used in this study was maceration of coffee beans before roasting. The fruit pulp contains high water content, which moisturizes the coffee beans, and together with solutes, this gives it a new, distinctive aroma. After extracting the beans from the fruit and before drying, it is beneficial to leave the beans in high humidity, where the germination can begin triggering the activity of different hydrolases. As a result, sugars, amino acids, and fatty acids are released, which are more reactive during drying and roasting than their precursors [22,23,24,25]. Using this method allows for obtaining coffee beans with lower acidity while maintaining high-quality green coffee beans. Therefore, dry-processed coffee was purchased for this research, so that the maceration would improve the properties of the beans.

Nowadays, many advanced methods are used to improve the aroma of coffee beans, including addition of volatile ingredients or their precursors depending on the stage of processing [26]. Initially, it involved enrichment with natural or artificial oils containing characteristic coffee aromas, enhancing the typical coffee smell. This practice continues to present day and applies to instant coffee. Later, the solutions that involved adding other aromas, such as vanilla, nut, chocolate, rum, cherry, and several others were developed [27]. However, coffee connoisseurs look for natural and more healthy solutions than flavoring by adding artificial substances. This has resulted in the emergence of other trends in improving the aroma of coffee and, at the same time, its health-promoting properties. These methods include removing harmful compounds and introducing aroma along with health-promoting substances, as well as maceration or fermentation using various methods [28,29,30,31]. The additional steps in processing coffee beans contribute to a higher final product price, making naturally flavored coffees a niche product so far.

For the fermentation of green coffee in fruit, and optionally also in extracted green beans, naturally inhibited coffee bush strains are used. By using microflora naturally adapted to the fermentation of the raw material, advanced fermentation can be achieved to obtain products such as coffee wine or coffee kumiss [32,33,34].

The content of caffeine, phenolic compounds and other highly bioactive substances may be changed because of natural favoring, which may influence the health-promoting properties of coffee extracts [35,36,37,38,39]. Evaluating the health-promoting properties of naturally flavored coffees may have the beneficial effect of increasing their consumption. Despite previous studies on coffee bean maceration in various media, there is no information in the literature on its effect on the level of hydroxycinnamic acids and their esters, of which coffee is an especially important source in the diet. Previous studies concerned volatile compounds and microbiological safety. Therefore, the novelty of this study is the assessment of the effect of coffee bean flavoring using different methods on the composition of polyphenolic compounds in the final product. The aim of this research was to assess the impact of coffee flavoring vs. maceration in different fruit pulps on properties of coffee extracts. It involved determination of the profile of phenolic and aliphatic acids, as well as antioxidant and α-amylase inhibitory properties.

## 2. Materials and Methods

### 2.1. Chemicals and Materials

HPLC- and UHPLC-grade water, formic acid, acetonitrile, methanol, hydroxy-2,5,7,8-tetramethylchroman-2-carboxylic acid (Trolox) (≥97%), 2,4,6-tri(2-pyridyl)-s-triazine (TPTZ, ≥98%), sodium acetate (≥99%), ferric chloride hexahydrate (≥97%), ferrozine (≥97%), 2,2-diphenyl-1-picrylhydrazyl (DPPH, ≥95%), disodium ethylenediaminetetraacetate dihydrate (EDTA, ACS reagent, 99.0–101.0%), and ammonium acetate (≥97%) were purchased from Merck (Darmstadt, Germany). Acrylamide (≥99%), 5-hydroxymethylfurfural (HMF, ≥99%), 5-caffeoylquinic acid (≥99%), lactic acid (≥98%), citric acid (≥98%), malic acid (≥99%), acetic 3,4-dicaffeoylquinic acid (≥98%), ferulic acid (FA, ≥98%), 5-Feruloylquinic acid (5FQ, ≥98%), 4-Feruloylquinic acid (4FQ, ≥98%), 3-Feruloylquinic acid (3FQ, ≥98%), α-amylase from human saliva, and caffeic acid (CA, ≥98%) were purchased from Sigma Aldrich (St. Louis, MO, USA); 3-caffeoylqunic acid (≥99%), 4-caffeoylqunic acid (≥99%), 3,5-dicaffeoylquinic acid (≥99%), and 4,5-dicaffeoylquinic acid (≥99%) were purchased from Phyto Lab (Vestenbergsgreuth, Germany). Ultrapure water (resistivity, 18.2 MΩ cm) was obtained from a Millipore Milli-Q Plus purification system (Bedford, MA, USA). Green Arabica, Brazil Cerrado variety (high-quality harvest, Brazil, Santos region) was purchased from Bero Polska (Gdynia, Poland). The coffee beans came from the 2018 harvest and were subjected to natural dry processing (drying in the sun and warm air). The fruits, food flavorings, and red wine were purchased from a local store.

### 2.2. Flavoring and Maceration of Coffee Beans

#### 2.2.1. Maceration in Wine

A quantity of 100 g green coffee was roasted at 230 °C for 12 min until the coffee beans were dark in color [40]. Obtaining a dark color (second crack) of the coffee ends the roasting process. The coffee beans were air-cooled. Freshly roasted coffee was cooled to 24 °C and poured with 200 mL of wine into a vessel, mixed, and closed. During maceration in wine, the coffee beans must be completely submerged to ensure that the coffee beans are evenly macerated; 200 mL was enough to immerse 100 g of green coffee beans. The maceration was carried out at a temperature of 4 °C for 12 h, with mixing of the coffee beans after 4 and 8 h. Then, the coffee beans were separated from the wine using a strainer. The procedure was repeated twice. A new portion of the same wine was used after 12 h to avoid the aftertaste of oxidized tannins. Maceration was carried out for a total of 24 h. Three parallel replicates were made from the same bottle of wine. The coffee beans were left on filter paper to evaporate the alcohol, and then the beans were dried at a temperature of 50 °C to ensure 95% solids content. Carlo Rossi (Bols Sp. z o.o., Oborniki, Poland), a sweet red wine (0.75 L, 9%) type with a floral and fruity aroma, was selected for analysis. The high sugar content in wine can cause reduced acidity in coffee. The wine was purchased at the supermarket. The bottles were selected from the same batch. For one-day fermentation, two new portions of wine from one bottle were used. According to the literature, wine fermentation can last longer than 2 days. For example, in the JP2017060455A patent, fermentation was carried out for 48 h and with the same pair of wine.

#### 2.2.2. Maceration in Fruit Pulps

**Figure 1:**
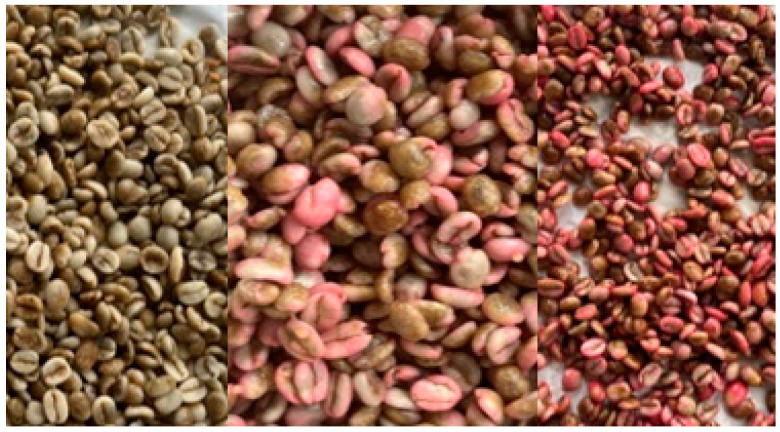
From the left: coffee macerated in melon, raspberry, strawberry.

Ripe, undamaged fruits were selected for analysis. The ripeness of the fruit was assessed organoleptically, by color and aroma. The amount of fruit to be macerated and the maceration time were selected experimentally, based on a preliminary study in which color and taste were assessed after various maceration times. Fresh apples, quince, melon, strawberry, raspberry, cherry, and grapefruit were washed and cut; 100 g of fruit, crushed using a kitchen grater and a mixer, was mixed with 100 g of green coffee beans. Coffee beans with fruit pulp were closed in a jar and placed in the refrigerator (4 °C) for 24 h. All variants were thoroughly mixed and placed in the refrigerator for a specified amount of time to carry out the maceration process. Then, the coffee beans were separated from the fruit under running water and dried for one day at a temperature of 50 °C to achieve 95% of solids. Next, 100 g of macerated green coffee was roasted at 230 °C for 12 min until the coffee beans were dark in color. The coffee beans were air-cooled. Figure 1 shows examples of coffee beans after maceration and drying.

#### 2.2.3. Flavoring with Aroma Oils

A quantity of 100 g green coffee beans was roasted at 230 °C for 12 min until the beans were dark in color [40]. The coffee beans were air-cooled. Freshly roasted coffee beans were mixed with 0.2 mL food flavoring in a coating machine. The operation was repeated for each flavor (mango, raspberry, grapefruit, strawberry, cherry, and vanilla).

#### 2.2.4. Preparation of Coffee Extracts

Coffee samples were ground in portions in a cast iron grinder (ground to a size range from 480 to 680 μm (Fiamma, MCF C)). Coffee infusion used for the analyses was prepared by placing 7 g of ground beans on filter paper (weight: 60 g/m^2^) on a funnel and pouring 100 mL of water at 95 °C.

### 2.3. Analysis of Phenolic and Organic Acid, Caffeine, HMF, and Acrylamide (UHPLC-ESI-MS)

**Figure 2:**
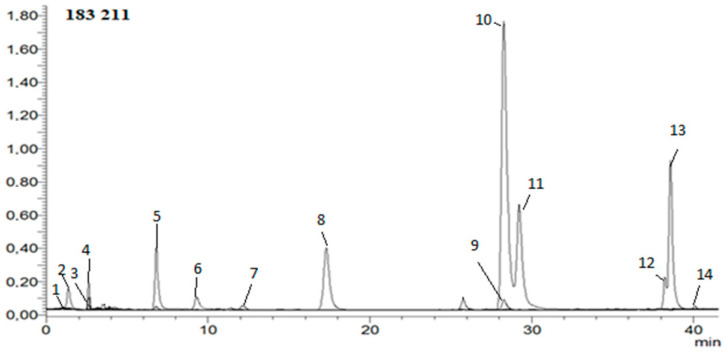
Example HPLC chromatogram of hydroxycinnamic acids, HMF, acrylamide, and caffeine in negative ion mode. 1—5-hydroxymethylfurfural; 2—acrylamide; 3—3-*O*-feruloylquinic acid; 4—4-*O*-feruloylquinic acid; 5—5-*O*-feruloylquinic acid; 6—ferulic acid; 7—caffeic acid; 8—4-*O*-caffeoylquinic acid; 9—3-*O*-caffeoylquinic acid; 10—caffeine; 11—5-*O*-caffeoylquinic acid; 12—3,4-di-*O*-caffeoylquinic acid; 13—3,5-di-O-caffeoylquinic acid; 14—4,5-di-*O*-caffeoylquinic acid.

Hydroxycinnamic, phenolic, and organic acids; caffeine; HMF; and acrylamide were determined in extracts. The analysis was carried out according to the method developed in the previous work [40]. In short: The mobile phase consisted of solvent A—water/formic acid (99.9:0.1, *v*/*v*); solvent B—acetonitrile/formic acid (99.9:0.1, *v*/*v*). A flow rate of 0.2 mL/min and injection volume of 2 μL was used. The calibration curves were constructed for a standard compound using six different concentration levels in a range of 0.01–1.0 mg/mL. Instrument control, data acquisition, and evaluation were carried out with LabSolutions 5.60 SP2, Chromatography Data System, and Postrun Analysis (LabSolutions 5.60 SP2) software, respectively. The mass spectrometric conditions were as follows: capillary voltage of 4500 V, drying gas temperature of 250 °C, drying gas flow of 15 L/min, and nebulizing gas pressure of 1 bar; the capillary temperature was 350 °C, and nitrogen was used as nebulizer. Full-scan mass spectra were acquired over a mass range from *m*/*z* 50 to 2000 in negative ion mode [40]. Figure 2 shows example HPLC chromatogram.

#### Determination of the Content of Organic Acids: Lactic, Citric, Malic, and Acetic

**Figure 3:**
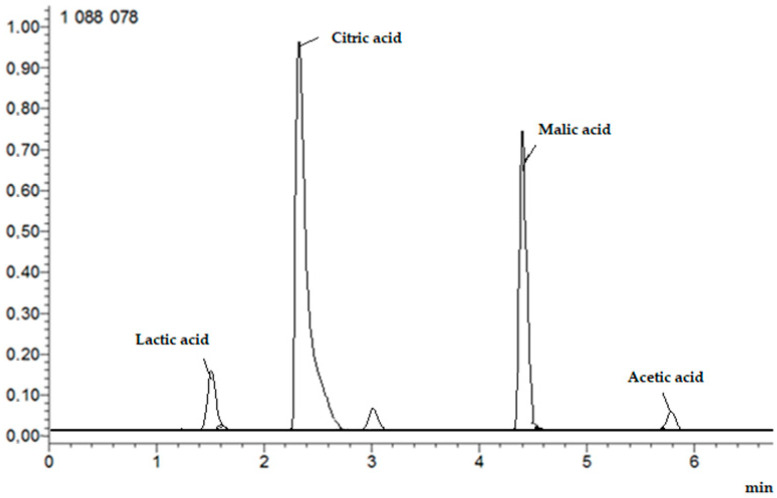
Example HPLC chromatogram of organic acids: lactic, citric, malic, and acetic.

The analysis was performed as above, with modifications. The mobile phases were eluent A, water/formic acid (99.9/0.1, *v*/*v*) and eluent B, methanol/formic acid (99.9/0.1, *v*/*v*). The flow rate was 0.3 mL/min, and the gradient was as follows: 0–1.30 min, 0–20% B; 1.31–3.00 min, 20–55% B; 3.01–6.00 min, 55–100% B; 6.01–7.00 min, 100–0% B. The ESI source was operated in positive ion mode and the column was thermostated at 35 °C. Full-scan MS and target MS2 spectral data were obtained from *m*/*z* 50 to 2000. Figure 3 shows example HPLC chromatogram.

### 2.4. Total Antioxidant Capacity Test

The total antioxidant capacity of the coffee was measured using spectrophotometric (Shimadzu, Kyoto, Japan); 0.2 g of ground coffee was extracted with 20 mL of methanol (70%) in ambient temperature for 30 min and filtered through filter paper. The filtrate was used for analyses. The antioxidant capacity was determined using the measurements of DPPH^•^ scavenging (0.1 mL of coffee extracts was added to 3.9 mL methanol and 1 mL DPPH; absorbance was measured at a wavelength of 517 nm) and of ferric reducing antioxidant power (FRAP) (1 mL of extracts was mixed with 4 mL of FRAP; absorbance was measured at a wavelength of 593 nm). The calibration was based on a reagent test without coffee extract. The DPPH^•^ and FRAP reducing activities were expressed as AC% = [(Acontrol − Asample)/Acontrol] × 100. The FRAP and DPPH were expressed in μmol TE/g [41,42].

### 2.5. α-Amylase Inhibitory Assay

The α-amylase inhibitory activity (AAI) was determined according to the method of Oboh [43]. α-Amylase from human saliva (A103) was dissolved in sodium phosphate buffer (pH 6.9; 20 mM) (1:9 *v*/*v*). Then, 500 µL of buffer and 500 µL of coffee extract prepared according to Section 2.5 were taken. The solution was mixed and incubated (25 °C). After 10 min, 500 µL of starch solution (1%) was added and the incubation was repeated under the same conditions. After 10 min, the incubation was stopped by adding 1.0 mL of dinitrosalicylic acid (DNSA) (5 min in boiling water bath). Then, the sample was cooled to room temperature and 10 mL of distilled water was added. Next, the absorbance was measured at 540 nm. The results were expressed as AAI inhibition (%) = [(Acontrol − Asample)/Acontrol] × 100.

### 2.6. Statistical Analysis

Statistical analysis was based on the determination of the average values of six measurements and their standard deviation, as well as on the one-way ANOVA (analysis of variance) and the Pearson correlation coefficient, using Statistic 10.0 software. The significance was defined at *p* ≤ 0.05.

## 3. Results and Discussion

### 3.1. Content of the Hydroxycinnamic and Organic Acid, Caffeine, HMF, and Acrylamide

The health-promoting properties of coffee extracts depend on the content of belonging to phenolic acids hydroxycinnamic acids (HCAs), in particular chlorogenic acid (CHAs). Different methods of refining coffee beans may change the content of HCAs [44,45]. Table 1 and Table 2 show the concentration of HCAs and caffeine in coffee extracts prepared from beans macerated in fruit pulps or in wine before roasting or with the addition of food flavors after roasting.

The content of HCAs ranged from 4.64 to 13.56 g/100 g d.b. for mango food flavor and melon pulp, respectively. The control sample (dark roasted coffee) showed an HCA content of 6.50 g/100 g d.b. The coffee bean maceration in fruit pulps resulted in an increase in the content of HCAs compared to the control (*p* < 0.05), in the following order: melon (13.56 g/100 g d.b.) > apples (13.22 g/100 g d.b.) > quinice (12.72 g/100 g d.b.) > cherry (10.80 g/100 g d.b.) >grapefruit (10.73 g/100 g d.b.) > raspberry (7.27 g/100 g d.b.). Maceration of coffee beans in strawberry pulp resulted in a statistically insignificant (*p* > 0.05) decrease in HCA content (Table 1). The reduction in HCAs in coffee beans may be due to the fact that strawberries are a rich source of flavonoids and hydroxybenzoic acids but not hydroxycinnamic acids [40]. Maceration in fruit pulps of raspberry, strawberry, cherry, and grapefruit resulted in a decrease of 5-*O*-caffeoylquinic acid concentration after roasting, which may be the result of partial transacylation of this isomer during thermal processing [46].

The most beneficial fruits used for coffee maceration in terms of HCA content increase were melon and apples. In both cases, the HCA concentration was approximately twice as high as in the control coffee extract (*p* < 0.05). The maceration resulted in an increase in the concentration of the isomers of ferulic acid esters with quinic acid (3-, 4-, and 5-O-feruloylquinic acids) and of caffeic acid esters (3- and 4-O-caffeoylquinic acids). However, the 5-O-caffeoylquinic acid isomer significantly increased its concentration only after maceration of coffee beans with melon, by approx. 30%. Dimtsas et al. showed that melon extracts contain 5-CQA acid at a level from 0.51 to 76.96 mg/100 g d.b and 3-CQA from 2.08 to 37.06 mg/100 g d.b. [47]. Melon is a rich source of polyphenols that prevent neurodegenerative diseases and diabetes. It exhibits antioxidant and anti-inflammatory properties. Additionally, it contains vitamins C and E. The main phenolic compounds in melon are chlorogenic acid (3-caffeoylquinic acid), neochlorogenic acid (5-caffeoylquinic acid), and 3-hydroxybenzoic acid. Therefore, during the maceration process, the mango pulp enriched the coffee with valuable HCAs (Table 1) [47,48,49].

Melon and apple have a high content of ascorbic acid. Ascorbic acid can protect coffee beans from the oxidation process during roasting and consequently yield higher amounts of HCA. Apples contain pectolytic enzymes that could cause an increase in chlorogenic acids as well because of the release from links with pectic substances. [47,48,49].

Caffeic and ferulic acids in roasted coffee were degraded compared to the control. The reduction may result from a high roasting temperature. For example, the thermostability of caffeic acid is up to 223 °C [50]. However, the acids may be additionally formed as a result of the esters’ bond hydrolysis, which is more likely at lower pH in the presence of fruit pulp substances. The feruloylquinic acids accounted for from 5 to 13% of the total chlorogenic acids. The decrease in determined trans-ferulic acid may also be the result of an increase in the content of ferulic acid cis-isomer [51].

The use of maceration in wine increased the HCA content by 0.94 g/100 g d.b. compared to the control sample (Table 2). However, the concentration achieved was not higher than in the case of fruit maceration. This may be related to the use of sweet wine, which could cause chlorogenic acid binding to the proteinaceous material and the formation of Maillard reaction products. In the case of wine-macerated coffee, an increase in 5-O-caffeoylquinic acid (Table 2) was also observed. Regarding polyphenols, coffee contains hydroxycinnamic acids and their esters, which account for about 10% of green beans and 3% of roasted coffee, and a cup of coffee contains 20–650 mg of these polyphenols. The wine is rich in flavan-3-ols, tannins, hydroxycinnamic and benzoic acids, anthocyanins, proanthocyanidins, and stilbenes, which include resveratrol, present in an amount of 3.5 mg/L [52]. Because of the rich and complex composition of wine, during coffee roasting after wine maturation, additional derivatives with unique sensory and health-promoting properties are formed [53]. Moderate drinking of wine and regular consumption of coffee improve cognitive effects in elderly people, inactivate cholinesterases, and provide neurotransmitters [54]. Polyphenols also show anticancer, cardioprotective, hepatoprotective, and immune system-stimulating properties, as well as anti-obesity and antidiabetic activities [55,56]. Phenolic compounds contained in coffee and wine have a beneficial effect on the gastrointestinal microbiota, which is reflected in the reduction in inflammatory and allergic reactions. The mechanism of action of polyphenols from both sources is different, and for this reason, it may be desirable to combine polyphenols from both sources in the diet, achieved with a specific product [57].

Enhancing the aroma of roasted coffee with the addition of food flavor contributed to reducing the content of HCAs. The mango food flavor reduced the HCA content by 1.86 g/100 g d.b., and by 1.21 g/100 g d.b. in the case of the raspberry flavor. However, the addition of strawberry, cherry, vanilla, and grapefruit food flavors had only a slight effect on the HCA reduction (Table 2). The differences resulted from the aroma carrier used. Strawberry, cherry, grapefruit, and vanilla flavors were dissolved in alcohol. However, mango and raspberry were dissolved in oils. Aromatic compounds in food flavors are dissolved in media such as ethyl alcohol, triacetin, propylene glycol, water, and vegetable oils. When adding flavor to hot coffee beans, the moisture content of the coffee beans may increase, which in turn could increase the acrylamide content [58]. On the other hand, food flavors can accelerate the oxidation process of roasted coffee beans, reducing the concentration of chlorogenic acids and lowering the overall quality of the brew [59].

Control coffee extracts, the extracts from beans roasted after maceration, and roasted bean extracts after the addition of aroma oils contained similar, statistically insignificant caffeine contents, amounting to about 3.5 g/100 g d.b. (*p* > 0.05). This was due to the fact that caffeine is a thermostable compound, and on the other hand, fruit pulps or aroma oils are not a source of alkaloids. Caffeine degrades at a temperature exceeding 235 °C [50]. Caffeine is an adenosine receptor inhibitor and has neuronal detoxification activity, influencing the release of glutamine and increasing the intracellular level of cyclic adenosine-3′,5′-monophosphate (cAMP). Caffeine is an antioxidant; in addition to inhibiting the action of adenosine, it helps to inhibit the activity of lipid peroxidase by degrading free radicals. Caffeine also has the ability to inhibit monoamine oxidase B, protecting and increasing the bioavailability of dopamine in the brain. Increasing dopamine concentration improves cognitive functions [60,61].

Maillard reaction products are an important group of coffee ingredients. Compounds from this group are attributed to quite different in vivo activities, ranging from anti-inflammatory and antioxidant activities to carcinogenic and mutagenic potential. HMF and acrylamide are examples of the second group. Table 3 shows the concentration of HMF and acrylamide in extracts from coffee macerated in fruit pulps or red wine as well as those flavored with aroma oils.

The HMF content in the extract from the control coffee was 0.33 mg/100 g d.b. (Table 3). The content of HMF in extracts after aromatization and maceration ranged from 0.30 to 1.34 mg/100 g for strawberry pulp and mango food flavor, respectively (Table 3). The maceration contributed to more intense formation of HMF using pulps obtained from melon, apple, raspberry, cherry, and grapefruit. As the fruits ripen, the sugar content increases, in particular sucrose, fructose, and galactosyl sucrose. Melon contains approximately 8 g/100 g; raspberry—4.42 g/100 g; cherry—5.95 g/100 g; grapefruit—7 g/100 g; apple—12.9 g/100 g of simple sugars. The higher the content of sugars, especially fructose, in the product, the higher the HMF content, which is consistent with our research. [62,63,64,65,66]. Most likely, some of the juice from the fruit was absorbed into the bean. A key issue in coffee research is the roasting process and its determining effect on the presence of biologically active phytochemicals. In this context, attention is mainly paid to the role of polyphenols, such as hydroxycinnamic acids and their esters with quinic acid, called chlorogenic acids, trigonelline, and Maillard reaction products (MRP) [67]. Thermal degradation of coffee bean components in the roasting process reduces the content of polyphenolic compounds and the formation of melanoidins, partly related to polyphenols, but also low molecular weight Maillard reaction products, such as acrylamide and 5-hydroxymethylfurfural, which reduce the antioxidant activity of coffee and have potential carcinogenic effects [67]. Already at a roasting temperature of 120–140 °C, an endothermic maximum can be observed; the coffee beans begin to swell, changing their structure. There are also processes of non-enzymatic bean browning, which contribute to the formation of acrylamide because of the reaction of the carboxyl group of reducing sugars with the amino group [68]. When coffee beans reach a temperature of approximately 160 °C, a characteristic crack appears, caused by a simultaneous loss of mass with the formation of gases and an increase in the volume of the bean [68]. At this stage of the coffee roasting process, changes in chemical composition occur, the degradation process of polyphenolic compounds begins, and a number of non-volatile organic compounds are transformed into volatile aroma components [68]. Pyrolysis of polysaccharides and sugars found in coffee beans takes place, producing volatile compounds, aliphatic carbonyls, alcohols, and diketones. At a temperature of 180–190 °C, the characteristic aroma, flavor, and color of light, medium, or dark roasted coffee are created. The reactions occurring in the grain at this temperature are exothermic, causing a rapid increase in grain temperature, leading to the degradation of proteins, chlorogenic acids, trigonelline, and carbohydrates [69]. Carbohydrates are degraded under the influence of aliphatic acids into smaller compounds, such as glycolic or lactic acid. As a result of non-enzymatic browning, because of the degradation of carbohydrates and chlorogenic acid, color-producing compounds such as melanoidins are formed [70,71]. The final stage of roasting coffee until it obtains a dark color is achieved at a bean temperature of 190 to 250 °C. The bean changes its structure, becomes highly porous, carbon dioxide is released, and new aromatic compounds and MRPs are formed, which partially combine with polyphenols [70,71].

Only strawberry and quince slightly reduced the content of HMF. This compound is formed because of the Maillard reaction or caramelization of sugars found in coffee beans [70]. At the same time, the formation of HMF is favored by a pH closer to neutral; therefore, sour strawberries and quince could limit its formation. Food flavors caused higher HMF content in coffee extracts than in the case of maceration because they contributed to increased non-enzymatic browning after adding them to coffee directly after roasting.

Acrylamide content in extracts from coffee macerated with fruit pulps or in wine was statistically at the same level (*p* > 0.05). However, the use of food flavors resulted in an increase in acrylamide to the range of 4.93–7.22 μg/100 g d.b. This may be related to the second possible mechanism of acrylamide formation from oxidized lipids.

Acrylamide consumed in high concentrations is a potentially carcinogenic substance, the permissible daily dose of acrylamide for a person weighing 70 kg amounts 140 μg. It can be noted that drinking coffee in quantities of 2–3 cups per day does not exceed the daily safe dose of acrylamide; however, this substance can be administrated additionally with other foods constituting the daily diet. It is known that consuming green coffee reduces the risk of acrylamide accumulation in the human body [72,73].

Our research confirmed that the addition of artificial flavors negatively affects the quality of the coffee infusion and its health-promoting properties. Food flavors contain partially natural plant and fruit extracts that contain simple sugars. This results in increased HMF and acrylamide. This is due to a reduction in the content of hydroxycinnamic acids and their esters in coffee extracts and the formation of a higher quantity of harmful compounds such as acrylamide. The content of some Maillard reaction products like HMF and acrylamide in coffee should be minimized, taking into account their anti-nutritional characteristics [74].

### 3.2. Concentration of Organic Acids

Roasted coffee beans subjected to the maceration in fruit pulps were richer in organic acids, except for apple fruit pulp. Apple pulp decreased organic acids by 0.02 g/100 g d.b. compared to the control sample. This is caused by a reduction in acetic acid and malic acid. Apples are a rich source of malic acid. However, the organic compound did not pass into the bean in sufficient quantity to compensate for the degradation of the compounds during coffee roasting [75]. Malic acid degrades at temperatures above 140 °C. A similar tendency of malic acid can be seen in raspberries and red wine. However, the content of malic acid increased in strawberries, melon, quince, cherries, and grapefruit. This may be related to the higher acidity in the fruit. Their concentrations increased from 0.87 to maximally 1.05 g/100 g d.b. in coffee macerated with melon pulp (Table 4). The contents of acetic and lactic acids increased in most variants. Enriching coffee beans with fruit pulp and wine resulted in an increase in the citric acid content in the beans, which at the same time had a protective effect on the citric acid present in the beans themselves. Citric acid degrades in the range of 177–501 °C, which makes it a stable compound [76]. A similar tendency was observed for lactic acid, which degrades only at a temperature of 185 °C [77].

The addition of food flavors contributed to reducing the content of organic acids by 0.07–0.14 g/100 g d.b., respectively, for vanilla and mango/grapefruit aroma oils (Table 4).

In the case of food aromas, the total content of organic acids decreased compared to the control sample. This may be due to the fact that coffee beans are sprinkled with food flavors right after roasting. A sticky layer is formed on the beans, which extends the cooling time of the beans. The consequence of this is the formation of a higher content of HMF and acrylamide, at the expense of acetic acid. Individual volatile compounds or groups of them are responsible for specific aroma characteristics. Lactic acid of low volatility and acetic acid result in a fruity and winey aroma in coffee [5,6,7,8,78]. In our study, coffee extracts after maceration with fruit pulps or red wine showed increased contents of lactic and acetic acids, which may give them fruitier aroma. Additionally, maceration with fruits may also result in the formation of an increased amount of furans and pyrroles, softening the taste of coffee [79,80]. This might result not only from absorption of phytochemicals from a pulp, because aromatic improvement was also observed after soaking green beans in water, followed by drying and roasting, which increased the content of free polyphenols and amino acids because of proteolysis [79,80].

### 3.3. Antioxidant Potential

**Figure 4:**
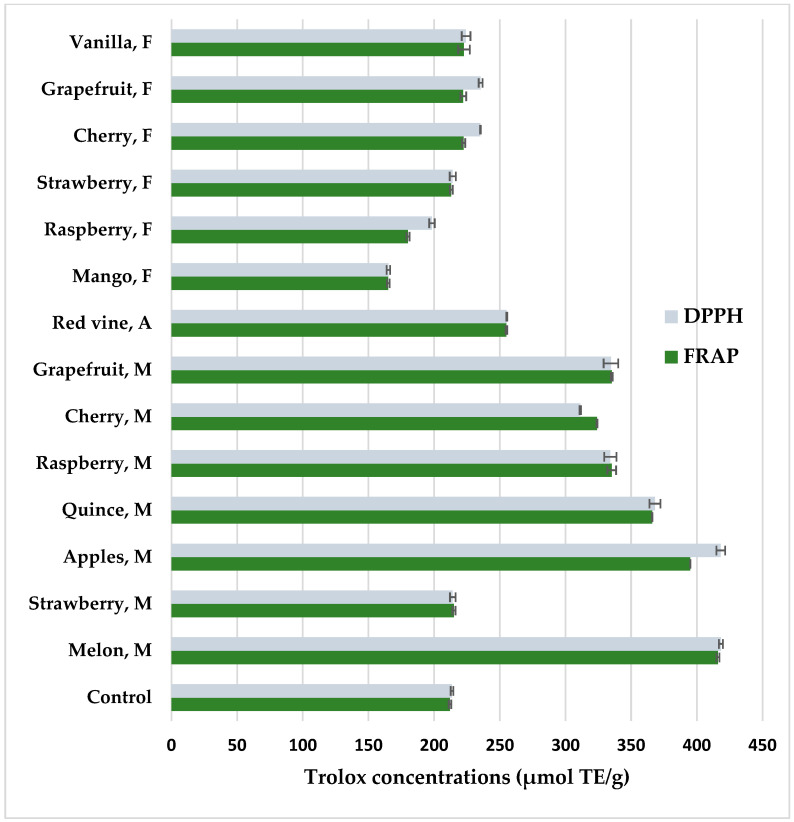
Antioxidant properties of methanolic extracts from flavored coffee, determined by DPPH and FRAP methods. Control—coffee without additives; M—maceration in fruit pulps; F—food flavors; A—maceration in red wine. Values are expressed as mean value ± SD.

Figure 4 shows antioxidant properties of coffee extracts prepared from coffee beans after maceration determined by DPPH and FRAP methods. Both methods showed a high positive correlation of the results, amounting to r = 0.98. Coffee methanolic extracts form the beans that had been pretreated by maceration with fruit pulps before roasting had increased antioxidant potential compared to the control (*p* < 0.05). Fruits such as melon (FRAP—416.77 μmol TE/g; DPPH—418.33 μmol TE/g), apple (FRAP—395.02 μmol TE/g; DPPH—418.22 μmol TE/g), and quince (FRAP—366.15 μmol TE/g; DPPH—368.12 μmol TE/g) showed the highest potential to increase the antioxidant activity in coffee. However, maceration in wine or the addition of food flavors such as cherry, strawberry, grapefruit, and vanilla resulted in similar antioxidant properties to the control coffee extract. In turn, mango and raspberry aroma reduced antioxidant properties. Melon has a high antioxidant potential because it is a source of vitamin C and A, β-carotene, and phytoene, with a total polyphenol content of 40–80 mg/100 g [80]. Maceration with the participation of hydrolytic enzymes releases sugars, amino acids, and polyphenols, which undergo non-enzymatic browning during roasting, so macerated coffees darken faster and show higher antioxidant activity after roasting. In addition to producing dark dyes, coffee fermentation contributes to enriching the aroma with a number of desirable compounds from the pyrazine, alcohol, aldehyde, ketone, phenol, furan, pyrrole, and other groups [81,82,83]. In fermented coffees, the content of organic acids such as malic and lactic increases, which give the infusions a pleasant acidity [84]. Maceration in strawberry pulp showed low antioxidant potential, which is related to the low content of chlorogenic acids. A better maceration effect could have been achieved with seasonal strawberries.

In turn, food flavors did not significantly increase the antioxidant potential of coffee extracts. In the case of mango aroma, there was a decrease in antioxidant activity, which was related to the decrease in HCA concentrations (Table 2). In food flavors, mango and cherry had the lowest value, which may be supplemented with more artificial additives to obtain the desired aroma. Some of the ingredients of food aromas with unsaturated bonds may create radicals with pro-oxidant action. The main antioxidant substances in coffee are chlorogenic acid and their volatile degradation products, caffeine, melanoidins, and trigonelline. It should be taken into account that coffee extracts are a rich source of antioxidants that are helpful in the prevention of, among others, Alzheimer’s and Parkinson’s diseases, type 2 diabetes, and metabolic orders [62,63].

### 3.4. α-Amylase Inhibitory Activity

**Figure 5:**
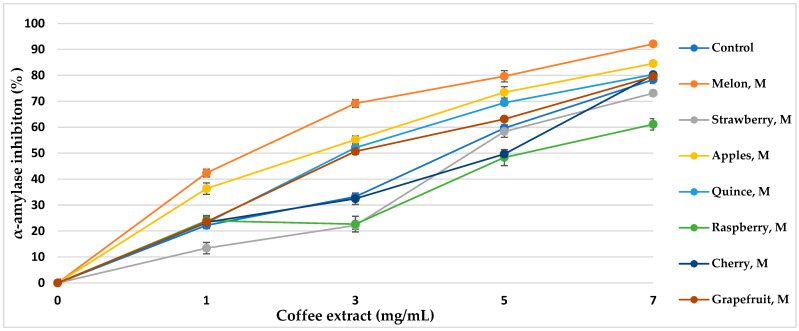
α-Amylase inhibitory activity of coffee extracts. Control—coffee without additives; M—maceration.

**Figure 6:**
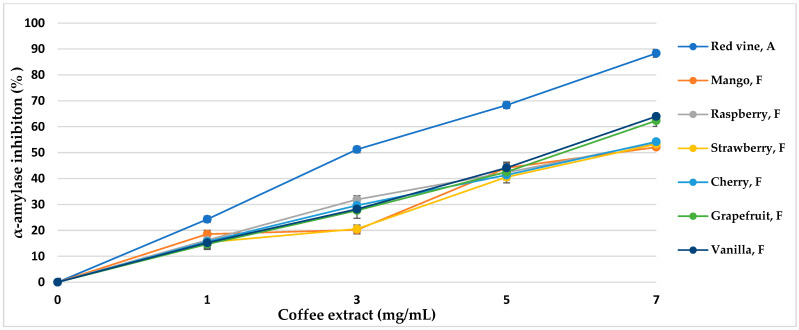
α-Amylase inhibitory activity of coffee extracts. Control—coffee without additives; F—food flavor; A—maceration in red wine.

α-Amylase is the main enzyme responsible for the decomposition of α-glucans by hydrolytic catalysis, making them bioavailable and potentially bioabsorbable. The use of α-amylase inhibitors is an essential element in the prevention of diabetes [43]. Coffee extracts subjected to various aromatization methods showed properties of over 50% inhibition of the enzyme (Table 5, Figure 5 and Figure 6). The inhibitory activity almost linearly increased with increasing concentrations of coffee extract in the tested range. The method of coffee bean maceration with fruit pulps of melon (92.11%, IC_50_ = 3.80 mg/mL), apple (84.55%, IC_50_ = 4.14 mg/mL), and red wine (88.33% IC_50_ = 3.96 mg/mL) showed the highest enzyme inhibition activity (α-amylase 0.5 mg/mL, 10 μmol/mL). However, the aromatization method with the addition of aroma oils from mango and raspberry showed enzyme inhibition activity lower than in the control sample.

Thanks to the maceration of coffee with fruits and wine, the activity of α-amylase inhibition increased, while the addition of food flavors generally decreased the mentioned inhibitory properties in coffee extracts. Sun et al. [85] showed that coffee treated with ultrasound before roasting increases the inhibitory effect towards α-amylase, and the compounds responsible for the activity bind the enzyme in a competitive manner. 5-*O*-Caffeoylquinic acid (5-CQA) and 3,5-*O*-dicaffeoylquinic acid (3,5-diCQA) were identified as the main coffee ligands linking the active site of α-amylase [85]. In our study, a similar relationship was observed. The extract of roasted coffee obtained after maceration in melon fruit pulp contained 5-CQA in relatively high amounts, at 5.76 g/100 g d.b., and at 1.34 g/100 g d.b. for 3,5-diCQA; the IC_50_ of the enzyme inhibition amounted to 3.80 mg/mL, and maceration in wine resulted in concentrations of 5.02 g/100 g d.b. and 0.46 g/100 g d.b., respectively, with IC_50_ = 3.96 mg/mL, comparing to other coffee aromatization methods and a control coffee. It can be noticed that maceration with fruit pulps not only increased the content of hydroxycinnamic acids and their esters in roasted coffee but also resulted in high alpha-amylase inhibition activity and antioxidant potential. However, food flavors showed low enzyme inhibition activity, which correlates with the low content of chlorophyll acids. Additionally, artificial flavors contain toxic substances and increased the content of acrylamide and HMF in the coffee extract, which resulted in lower antioxidant activity.

## 4. Conclusions

This study showed that the content of phenolic compounds and organic acids in coffee extracts depended on the coffee flavoring method. It was found that maceration of green coffee beans with mango, apple, and red wine is the best way to flavor coffee in terms of health-promoting properties. Maceration had a positive effect on the protection of beans against the degradation of phenolic compounds. Roasted coffee with added food flavors had the lowest content of polyphenols and organic acids, which resulted in a deterioration in quality. The obtained results suggest a beneficial effect of using fruit pulps or wine for green bean maceration on antioxidant and α-amylase-inhibiting activity. The results of this study will be used in future research on an innovative method of coffee bean refining, optimizing maceration conditions using wastes from the food industry, the aim of which is not only to improve the taste but also to have a beneficial effect on the health-promoting properties of coffee beans, evaluated in complex in vitro and in vivo models.

## Figures and Tables

**Table 1:** Concentration of hydroxycinnamic and caffeine in extracts from coffee macerated in fruit pulps and roasted. CA—caffeic acid; FA—ferulic acid; 3-FQA—3-*O*-Feruloylquinic acid; 4-FQA—4-*O*-Feruloylquinic acid; 5-FQA—5-*O*-Feruloylquinic acid; 3-CQA—3-*O*-Caffeoylquinic acid; 4-CQA—4-*O*-Caffeoylquinic acid; 5-CQA—5-*O*-Caffeoylquinic acid; 3,5-diCQA—3,5-*O*-Dicaffeoylquinic acid; 4,5-diCQA—4,5-*O*-Dicaffeoylquinic acid; 3,4-diCQA—3,4-*O*-Dicaffeoylquinic acid; TCA—total chlorogenic acids.

Compound (g/100 g d.b.)	Control	Melon	Strawberry	Apples	Quince	Raspberry	Cherry	Grapefruit
CA	0.13 ± 0.01 ^a^	0.08 ± 0.00 ^b^	0.12 ± 0.01 ^a,c^	0.06 ± 0.00 ^b^	0.05 ± 0.00 ^b^	0.12 ± 0.00 ^a,c^	0.10 ± 0.00 ^a^	0.07 ± 0.00 ^b^
FA	0.36 ± 0.03 ^a^	0.21 ± 0.00 ^b^	0.35 ± 0.00 ^a^	0.26 ± 0.00 ^b^	0.28 ± 0.00 ^b^	0.36 ± 0.01 ^a^	0.22 ± 0.00 ^b^	0.21 ± 0.01 ^b^
3-FQA	0.09 ± 0.02 ^a^	0.31 ± 0.00 ^b^	0.11 ± 0.00 ^a^	0.37 ± 0.01 ^c^	0.39 ± 0.01 ^c^	0.10 ± 0.00 ^a^	0.31 ± 0.01 ^b^	0.28 ± 0.01 ^d^
5-FQA	0.33 ± 0.01 ^a^	0.82 ± 0.02 ^b^	0.35 ± 0.00 ^a^	1.10 ± 0.02 ^c^	1.15 ± 0.02 ^c^	0.48 ± 0.01 ^d^	0.88 ± 0.02 ^b^	0.95 ± 0.01 ^e^
4-FQA	0.09 ± 0.00 ^a^	0.40 ± 0.00 ^c^	0.09 ± 0.00 ^a^	0.45 ± 0.00 ^c^	0.43 ± 0.00 ^c^	0.11 ± 0.00 ^a^	0.42 ± 0.00 ^c^	0.42 ± 0.00 ^c^
3-CQA	0.38 ± 0.01 ^a^	1.82 ± 0.01 ^b^	0.43 ± 0.01 ^c^	1.38 ± 0.01 ^d^	1.41 ± 0.01 ^d^	0.73 ± 0.03 ^e^	1.96 ± 0.04 ^b^	1.20 ± 0.02 ^d^
5-CQA	4.25 ± 0.03 ^a^	5.71 ± 0.03 ^c^	4.02 ± 0.01 ^a^	4.35 ± 0.61 ^b^	4.33 ± 0.61 ^b^	4.15 ± 0.02 ^a^	3.18 ± 0.15 ^c^	2.88 ± 0.03 ^d^
4-CQA	0.38 ± 0.02 ^a^	1.79 ± 0.02 ^b^	0.36 ± 0.01 ^a^	1.98 ± 0.06 ^b^	2.01 ± 0.06 ^c^	0.44 ± 0.01 ^d^	1.51 ± 0.05 ^e^	1.63 ± 0.01 ^e^
3,5-diCQA	0.49 ± 0.02 ^a^	1.34 ± 0.01 ^c^	0.46 ± 0.01 ^a^	1.39 ± 0.07 ^c^	0.92 ± 0.07 ^b^	0.45 ± 0.01 ^a^	1.17 ± 0.01 ^c^	1.95 ± 0.02 ^d^
4,5-diCQA	0.04 ± 0.00 ^a^	0.21 ± 0.01 ^b^	0.03 ± 0.01 ^a^	0.32 ± 0.00 ^c^	0.35 ± 0.00 ^c^	0.02 ± 0.00 ^a^	0.22 ± 0.01 ^b,d^	0.22 ± 0.00 ^d,b^
3,4-diCQA	0.11 ± 0.00 ^a^	0.87 ± 0.01 ^b^	0.10 ± 0.00 ^a^	1.56 ± 0.03 ^c^	1.58 ± 0.03 ^c^	0.31 ± 0.01 ^d^	0.83 ± 0.01 ^b^	0.92 ± 0.01 ^b^
TCA	6.50 ± 0.09 ^a^	13.56 ± 0.05 ^c^	6.42 ± 0.03 ^a^	13.22 ± 0.45 ^c^	12.72 ± 0.45 ^b^	7.27 ± 0.09 ^d^	10.80 ± 0.29 ^e^	10.73 ± 0.15 ^e^
Caffeine	3.51 ± 0.03 ^a^	3.50 ± 0.01 ^a^	3.49 ± 0.02 ^a^	3.51 ± 0.01 ^a^	3.51 ± 0.03 ^a^	3.50 ± 0.02 ^a^	3.50 ± 0.02 ^a^	3.52 ± 0.01 ^a^

Values are expressed as mean value ± SD; *n* = 6; values with the same superscript letter (^a–e^) along the same row are not significantly different (*p* > 0.05).

**Table 2:** Concentration of hydroxycinnamic and caffeine in in extracts from coffee macerated in red wine and roasted or flavored with aroma oils after roasting. CA—caffeic acid; FA—ferulic acid; 3-FQA—3-*O*-Feruloylquinic acid; 4-FQA—4-*O*-Feruloylquinic acid; 5-FQA—5-*O*-Feruloylquinic acid; 3-CQA—3-*O*-Caffeoylquinic acid; 4-CQA—4-*O*-Caffeoylquinic acid; 5-CQA—5-*O*-Caffeoylquinic acid; 3,5-diCQA—3,5-*O*-Dicaffeoylquinic acid; 4,5-diCQA—4,5-*O*-Dicaffeoylquinic acid; 3,4-diCQA—3,4-*O*-Dicaffeoylquinic acid; TCA—total chlorogenic acids.

Compound (g/100 g d.b.)	Mango	Raspberry	Strawberry	Cherry	Grapefruit	Vanilla	Red Wine
CA	0.08 ± 0.00 ^b^	0.09 ± 0.00 ^b^	0.07 ± 0.00 ^b^	0.06 ± 0.00 ^c^	0.08 ± 0.00 ^b^	0.06 ± 0.00 ^c^	0.12 ± 0.00 ^a^
FA	0.12 ± 0.01 ^b^	0.23 ± 0.01 ^d^	0.19 ± 0.00 ^a^	0.17 ± 0.01 ^c^	0.23 ± 0.01 ^d^	0.17 ± 0.00 ^c^	0.35 ± 0.02 ^a^
3-FQA	0.21 ± 0.01 ^d^	0.33 ± 0.01 ^c^	0.26 ± 0.01 ^d^	0.13 ± 0.01 ^b^	0.32 ± 0.03 ^c^	0.23 ± 0.01 ^d^	0.11 ± 0.00 ^a^
5-FQA	0.15 ± 0.02 ^b^	0.25 ± 0.02 ^c^	0.29 ± 0.01 ^d^	0.20 ± 0.01 ^c^	0.26 ± 0.02 ^c^	0.70 ± 0.02 ^e^	0.37 ± 0.01 ^a^
4-FQA	0.19 ± 0.02 ^b^	0.40 ± 0.01 ^c^	0.32 ± 0.00 ^d^	0.29 ± 0.00 ^e^	0.20 ± 0.02 ^b^	0.29 ± 0.03 ^e^	0.09 ± 0.00 ^a^
3-CQA	0.47 ± 0.03 ^c^	0.06 ± 0.02 ^b^	0.63 ± 0.01 ^d^	0.46 ± 0.02 ^c^	0.50 ± 0.16 ^e^	0.46 ± 0.03 ^c^	0.43 ± 0.01 ^c^
5-CQA	2.39 ± 0.05 ^b^	2.79 ± 0.09 ^b^	3.80 ± 0.03 ^c^	3.38 ± 0.02 ^c^	3.65 ± 0.13 ^c^	2.38 ± 0.01 ^b^	5.02 ± 0.02 ^d^
4-CQA	0.26 ± 0.05 ^b^	0.51 ± 0.05 ^c^	0.15 ± 0.03 ^d^	0.61 ± 0.00 ^e^	0.34 ± 0.03 ^a^	0.91 ± 0.01 ^f^	0.36 ± 0.01 ^a^
3,5-diCQA	0.14 ± 0.02 ^a^	0.33 ± 0.03 ^c^	0.29 ± 0.03 ^d^	0.15 ± 0.01 ^a^	0.28 ± 0.02 ^f^	0.15 ± 0.02 ^a^	0.46 ± 0.02 ^f^
4,5-diCQA	0.16 ± 0.02 ^b^	0.21 ± 0.02 ^c^	0.22 ± 0.02 ^c^	0.20 ± 0.00 ^c^	0.26 ± 0.01 ^c^	0.19 ± 0.01 ^b^	0.03 ± 0.00 ^a^
3,4-diCQA	0.13 ± 0.01 ^a^	0.20 ± 0.02 ^d^	0.27 ± 0.01 ^d^	0.75 ± 0.02 ^b^	0.30 ± 0.05 ^c^	0.92 ± 0.03 ^d^	0.10 ± 0.00 ^a^
TCA	4.64 ± 0.15 ^b^	5.29 ± 0.10 ^c^	6.28 ± 0.03 ^a^	6.48 ± 0.09 ^a^	6.41 ± 0.03 ^d^	6.44 ± 0.15 ^a^	7.44 ± 0.15 ^d^
Caffeine	3.50 ± 0.01 ^a^	3.50 ± 0.02 ^a^	3.49 ± 0.01 ^a^	3.41 ± 0.02 ^a^	3.50 ± 0.04 ^a^	3.50 ± 0.02 ^a^	3.51 ± 0.00 ^a^

Values are expressed as mean value ± SD; *n* = 6; values with the same superscript letter (^a–f^) along the same row are not significantly different (*p* > 0.05).

**Table 3:** Concentration of HMF and acrylamide in extracts from coffee macerated in fruit pulps, red wine, or flavored with aroma oils.

Coffee Handling Method	5-Hydroxymethylfurfural (mg/100 g d.b.)	Acrylamide (μg/100 g d.b.)
Control	0.33 ± 0.01 ^a^	4.89 ± 0.06 ^a^
Maceration		
Melon	0.44 ± 0.02 ^b^	4.90 ± 0.03 ^a^
Strawberry	0.30 ± 0.01 ^a^	4.91 ± 0.02 ^a^
Apples	0.55 ± 0.03 ^c^	4.98 ± 0.09 ^a^
Quince	0.31 ± 0.01 ^a^	5.01 ± 0.03 ^b^
Raspberry	0.38 ± 0.02 ^a^	4.95 ± 0.02 ^a^
Cherry	0.35 ± 0.02 ^a^	4.91 ± 0.04 ^a^
Grapefruit	0.41 ± 0.03 ^b^	4.90 ± 0.01 ^a^
Red vine	0.35 ± 0.02 ^a^	4.91 ± 0.01 ^a^
Food flavors		
Mango	1.34 ± 0.01 ^d^	6.95 ± 0.06 ^a^
Raspberry	1.22 ± 0.01 ^d^	6.33 ± 0.09 ^c^
Strawberry	1.05 ± 0.02 ^d^	7.22 ± 0.11 ^d^
Cherry	0.95 ± 0.03 ^c^	5.51 ± 0.02 ^b^
Grapefruit	0.98 ± 0.04 ^c^	5.18 ± 0.04 ^b^
Vanilla	0.68 ± 0.03 ^e^	4.93 ± 0.01 ^a^

Values are expressed as mean value ± SD; *n* = 6. Values with the same superscript letter (^a–e^) along the same row are not significantly different (*p* > 0.05).

**Table 4:** Concentration of organic acids in extracts from coffee macerated in fruit pulps, red wine, or flavored with aroma oils.

Coffee Handling Method	Acetic Acid (mg/100 g d.b.)	Lactic Acid (mg/100 g d.b.)	Malic Acid (g/100 g d.b.)	Citric Acid (g/100 g d.b.)	Total Organic Acids (g/100 g d.b.)
Control	0.72 ± 0.02 ^a^	3.23 ± 0.01 ^a^	0.25 ± 0.02 ^a^	0.61 ± 0.01 ^a^	0.87 ± 0.03 ^a^
Maceration					
Melon	1.24 ± 0.01 ^b^	4.16 ± 0.12 ^b^	0.29 ± 0.04 ^b^	0.71 ± 0.05 ^b^	1.05 ± 0.05 ^b^
Strawberry	1.44 ± 0.01 ^c^	7.05 ± 0.05 ^c^	0.29 ± 0.01 ^b^	0.65 ± 0.07 ^c^	0.95 ± 0.09 ^c^
Apples	0.22 ± 0.01 ^d^	3.71 ± 0.11 ^d^	0.20 ± 0.03 ^c^	0.65 ± 0.02 ^c^	0.85 ± 0.05 ^a^
Quince	0.63 ± 0.00 ^e^	4.89 ± 0.03 ^e^	0.28 ± 0.02 ^b^	0.66 ± 0.01 ^c^	0.94 ± 0.03 ^c^
Raspberry	1.22 ± 0.01 ^b^	4.72 ± 0.10 ^e^	0.23 ± 0.04 ^a^	0.65 ± 0.02 ^c^	0.89 ± 0.04 ^a^
Cherry	1.59 ± 0.02 ^c^	4.66 ± 0.05 ^e^	0.27 ± 0.02 ^b^	0.63 ± 0.05 ^a^	0.91 ± 0.02 ^c^
Grapefruit	1.61 ± 0.02 ^c^	3.73 ± 0.09 ^d^	0.26 ± 0.03 ^a^	0.65 ± 0.04 ^c^	0.93 ± 0.02 ^c^
Red vine	1.13 ± 0.06 ^b^	3.76 ± 0.09 ^d^	0.23 ± 0.03 ^c^	0.76 ± 0.02 ^b^	0.99 ± 0.09 ^c^
Food flavors					
Mango	0.04 ± 0.00 ^f^	0.01 ± 0.00 ^f^	0.27 ± 0.01 ^b^	0.46 ± 0.02 ^d^	0.73 ± 0.02 ^d^
Raspberry	0.05 ± 0.01 ^f^	0.01 ± 0.00 ^f^	0.23 ± 0.02 ^c^	0.55 ± 0.02 ^e^	0.78 ± 0.03 ^d^
Strawberry	0.19 ± 0.01 ^g^	0.01 ± 0.00 ^f^	0.20 ± 0.02 ^d^	0.58 ± 0.03 ^e^	0.78 ± 0.03 ^d^
Cherry	0.16 ± 0.01 ^g^	0.01 ± 0.00 ^f^	0.21 ± 0.05 ^d^	0.58 ± 0.08 ^e^	0.79 ± 0.02 ^d^
Grapefruit	0.22 ± 0.01 ^h^	0.01 ± 0.00 ^f^	0.16 ± 0.03 ^e^	0.57 ± 0.02 ^e^	0.73 ± 0.03 ^d^
Vanilla	0.02 ± 0.00 ^f^	0.01 ± 0.00 ^f^	0.21 ± 0.02 ^d^	0.59 ± 0.0 1 ^e^	0.80 ± 0.03 ^e^

Values are expressed as mean value ± SD; *n* = 6. Values with the same superscript letter (^a–h^) along the same row are not significantly different (*p* > 0.05).

**Table 5:** IC_50_ values of α- amylase inhibitory activity of coffee extract form flavored beans.

IC_50_ (mg/mL) *							
Control	Maceration						
	Melon	Strawberry	Apples	Quince	Raspberry	Cherry	Grapefruit
4.47 ± 0.05 ^a^	3.80 ± 0.15 ^b^	4.79 ± 0.09 ^a^	4.14 ± 0.03 ^a^	4.36 ± 0.29 ^a^	5.73 ± 0.15 ^c^	4.37 ± 0.18 ^a^	4.40 ± 0.07 ^a^
Food flavors						Maceration	
Mango	Raspberry	Strawberry	Cherry	Grapefruit	Vanilla	Red wine	
6.72 ± 0.34 ^d^	6.50 ± 0.09 ^d^	6.56 ± 0.02 ^d^	6.46 ± 0.10 ^d^	5.62 ± 0.13 ^c^	5.47 ± 0.05 ^c^	3.96 ± 0.35 ^b^	

Values are expressed as mean value ± SD; *n* = 6. Values with the same letter ^a–d^ along the same column are not significantly different (*p* > 0.05). * IC_50_—the half-maximal inhibitory concentration values for α-amylase by water-extracted coffee.

## Data Availability

The data presented in this study are available on request from the corresponding author due to he data are part of an ongoing study.
